# The Effect of the Idiopathic Epiretinal Membrane and Surgically Induced Posterior Vitreous Detachment on the Retinal Nerve Fiber Layer

**DOI:** 10.1155/2020/5217645

**Published:** 2020-11-18

**Authors:** A. Altun

**Affiliations:** Bahçeşehir University, Department of Ophthalmology, Istanbul, Turkey

## Abstract

**Aim:**

To investigate the changes in the retinal nerve fiber layer (RNFL) following pars plana vitrectomy (PPV) with surgically induced posterior vitreous detachment (PVD) and idiopathic epiretinal membrane (ERM) and internal limiting membrane (ILM) peeling.

**Methods:**

Patients with unilateral ERM with vitreomacular traction were included in this prospective, randomized, and controlled clinical trial. The control group (Group 1) was formed with the nonoperated fellow eyes of the patients, and the study group (Group 2) was formed with the eyes that underwent PPV including idiopathic ERM and ILM peeling. In the preoperative and postoperative periods (1^st^, 2^nd^, 3^rd^, 6^th^, and 12^th^ months), complete ophthalmological examination of the eyes was performed and RNFL measurements were examined in 4 different quadrants (superior, temporal, inferior, and nasal) with the help of spectral domain optical coherence tomography (OCT).

**Results:**

There was no statistically significant change in Group 1 during the follow-up period in all quadrants (*p* > 0.05). The mean RNFL thickness in Group 2 was statistically significantly higher than in Group 1 in superior, inferior, and temporal quadrants (*p* < 0.01), preoperatively. The mean RNFL in Group 2 was higher in the 1^st^, 2^nd^, 3^rd^, and 6^th^ months and lower in the 12^th^ month in superior, inferior, and temporal quadrants (*p* < 0.01) when compared to the preoperative period. The mean RNFL thickness in the nasal quadrant in Group 2 was higher in the 1^st^, 2^nd^, and 3^rd^ (*p* < 0.01) months, same in the 6^th^ month (*p* > 0.05), and lower in the 12^th^ (*p* < 0.01) month when compared to the preoperative period.

**Conclusion:**

Idiopathic ERM may cause an increase in RNFL thickness in superior, inferior, and temporal quadrants with possible tractional effect. PPV with PVD induction and ERM and ILM peeling may cause these RNFL changes.

## 1. Introduction

The retinal nerve fiber layer (RNFL) mainly consists of axons of the ganglion cells in the retina and transmits the visual stimulus to the brain by forming the optic nerve [1]. Many factors and diseases that could change RNFL thickness have been described in the literature. In addition to diseases that directly affect the optic nerve and glaucoma, central retinal vein occlusion [2], multiple sclerosis [3], migraine [4], Alzheimer's disease [5, 6], and diabetic retinopathy [7] are some of them.

It has been reported that ERM and PPV may also cause changes in the RNFL [8–11]. In this prospective, controlled clinical study, we aimed to investigate the effect of the idiopathic epiretinal membrane (ERM) and surgically induced PVD on RNFL in patients that underwent pars plana vitrectomy (PPV).

## 2. Methods

This prospective, randomized, and controlled clinical study was approved by the Institutional Ethics Committee of University of Health Sciences (Study number: 17073117-050.99-2909) and was performed in accordance with the tenets of the Declaration of Helsinki. Patients who applied to our clinic between January 2018 and January 2019 with symptoms associated with unilateral idiopathic ERM and that underwent 25-gauge PPV operation were included in this study. The control group (Group 1) was formed with healthy fellow eyes of the patients with the absence of ERM on OCT imagining. The study group (Group 2) was formed with pseudophakic eyes of the patients that underwent ERM and ILM peeling during PPV. Cases with PVD, glaucoma, migraine, Azheimer's disease, cerebrovascular disease, multiple sclerosis, pregnancy, history of intraocular surgery, macular or retinal disease other than idiopathic ERM, crystalline lens, diabetes mellitus, amblyopia, any kind of optic neuropathy, corneal pathology, and uveitis were excluded from the study.

The presence or absence of PVD was determined by B-scan ultrasonography, funduscopic examination, and spectral domain optical coherence tomography (OCT) (Topcon 3D OCT 2000FA, Tokio, Japan). During vitreoretinal surgery, ILM was dyed with brillant blue G (BBG) to increase visibility in eyes in Group 1. BBG with the concentration of 0.05 mg/mL was applied for 2 seconds on the macula. No dye or triamcinolone was used to increase vitreous visibility in any eyes during PPV. The same experienced vitreoretinal surgeon (AA) using the same instrument (Constellation, Alcon, USA) performed 25-gauge sutureless transconjunctival PPV operation with the help of retrobulbar anesthesia. Endotamponade (air, gas, and oil) implantation was not performed in any case.

Full ophthalmologic examination and OCT imaging were performed in the one-year follow-up period. RNFL follow-up was conducted preoperatively and postoperatively at 1^st^, 2^nd^, 3^rd^, 6^th^, 9^th^, and 12^th^ months. OCT imaging was performed through undilated pupils and with the same intensity of dim room lighting. All images were taken by the same operatör and evaluated by the same retina specialist (AA). The peripapillary RNFL was evaluated separately in superior, inferior, nasal, and temporal quadrants. The best corrected visual acuity (BCVA) of the eyes was determined with the Snellen chart and was converted to log MAR for statistical analysis. The distribution of variables is measured by the Kolmogorov Smirnov test. The Wilcoxon test was used in the analysis of dependent quantitative data. SPSS 26.0 program was used in the analysis, and the significance level was set at less than 0.05.

## 3. Results

A total of 64 eyes of 32 patients were included in this study. All patients had Caucasian ethnicity. The mean age of the patients was 59.5 ± 5.6 years, and the female : male ratio was 36 : 28. The mean BCVA level in the preoperative period was 0.03 ± 0.04 and 0.80 ± 0.32 LogMAR in Group 1 and Group 2, respectively. At the last examination after twelve months, the mean BCVA level improved to 0.03 ± 0.23 ± 0.10 LogMAR in Group 2. The mean BCVA level of vitrectomized eyes in Group 2 improved statistically significantly compared to the preoperative period (*p* < 0.01). There was no development of late-onset ocular hypertension (LOH) in any of the eyes included in the study.

**Figure 1:**
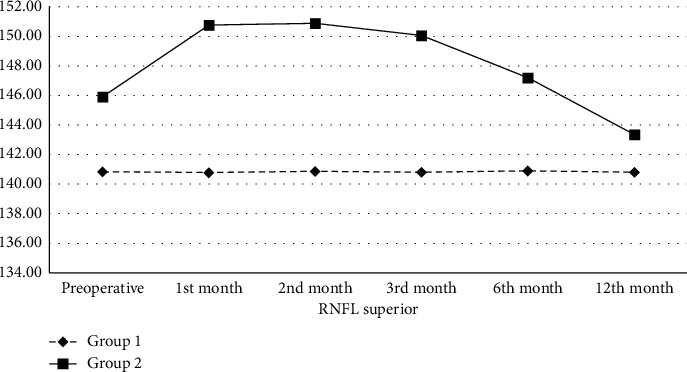
Retinal nerve fiber layer thickness (*μ*m) change in the superior quadrant of the groups. RNFL: retinal nerve fiber layer; *μ*m: micrometer.

In the preoperative period, the mean RNFL thickness in the superior quadrant in Group 1 and Group 2 was 140.85 ± 5.86 and 145.89 ± 5.88 *μ*m, respectively. The mean RNFL thickness in the superior quadrant in Group 2 was statistically significantly higher than in Group 1 preoperatively (*p* < 0.01). While there was no statistically significant change in Group 1 during the follow-up period, the mean RNFL thickness in the superior quadrant in Group 2 compared to the preoperative period was higher in the 1^st^ (150.76 ± 6.31 *μ*m, *p* < 0.01), 2^nd^ (150.88 ± 6.38 *μ*m, *p* < 0.01), 3^rd^ (150.05 ± 6.30 *μ*m, *p* < 0.01), and 6^th^ (147.20 ± 6.83 *μ*m, *p* < 0.01) months and lower in the 12^th^ (143.35 ± 7.13 *μ*m, *p* < 0.01) month (Table 1, Figure 1).

**Figure 2:**
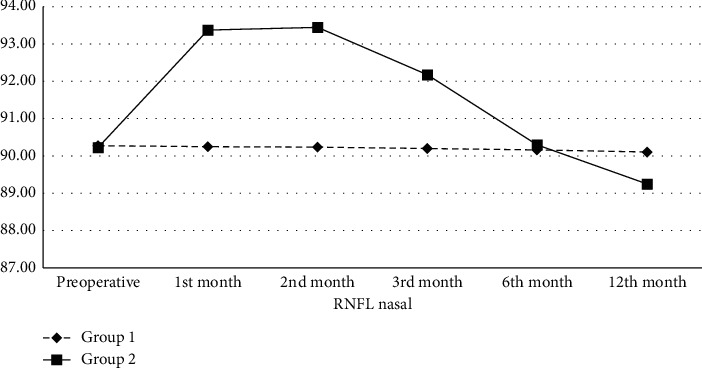
Retinal nerve fiber layer thickness (*μ*m) change in the nasal quadrant of the groups. RNFL: retinal nerve fiber layer; *μ*m: micrometer.

In the preoperative period, the mean RNFL thickness in the nasal quadrant in Group 1 and Group 2 was 90.27 ± 4.69 and 90.22 ± 4.43 *μ*m, respectively. There was no statistically significant difference between the groups in terms of RNFL thickness in the nasal quadrant in the preoperative period (*p* = 0.810). While there was no statistically significant change in Group 1 during the follow-up period, the mean RNFL thickness in the nasal quadrant in Group 2 compared to the preoperative period was higher in the 1^st^ (93.37 ± 4.60 *μ*m, *p* < 0.01), 2^nd^ (93.44 ± 4.60 *μ*m, *p* < 0.01), and 3^rd^ (92.18 ± 4.60 *μ*m, *p* < 0.01) months, similar in the 6^th^ month (90.30 ± 4.38 *μ*m, *p* = 0.695), and lower in the 12^th^ (89.25 ± 4.38 *μ*m, *p* < 0.01) month (Table 2, Figure 2).

**Figure 3:**
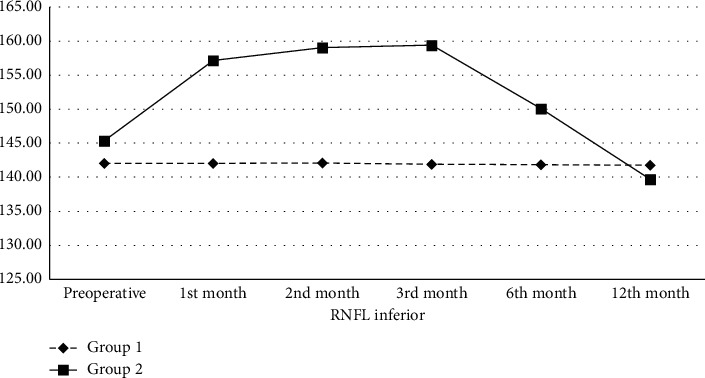
Retinal nerve fiber layer thickness (*μ*m) change in the inferior quadrant of the groups. RNFL: retinal nerve fiber layer; *μ*m: micrometer.

In the preoperative period, the mean RNFL thickness in the inferior quadrant in Group 1 and Group 2 was 142.03 ± 6.26 and 145.36 ± 6.11 *μ*m, respectively. The mean RNFL thickness in the inferior quadrant in Group 2 was statistically significantly higher than in Group 1 preoperatively (*p* < 0.01). While there was no statistically significant change in Group 1 during the follow-up period, the mean RNFL thickness in the inferior quadrant in Group 2 compared to the preoperative period was higher in the 1^st^ (157.15 ± 6.95 *μ*m, *p* < 0.01), 2^nd^ (159.01 ± 7.13 *μ*m, *p* < 0.01), 3^rd^ (159.41 ± 7.10 *μ*m, *p* < 0.01), and 6^th^ (150.10 ± 8.48 *μ*m, *p* < 0.01) months and lower in the 12^th^ (139.67 ± 6.96 *μ*m, *p* < 0.01) month (Table 3, Figure 3).

**Figure 4:**
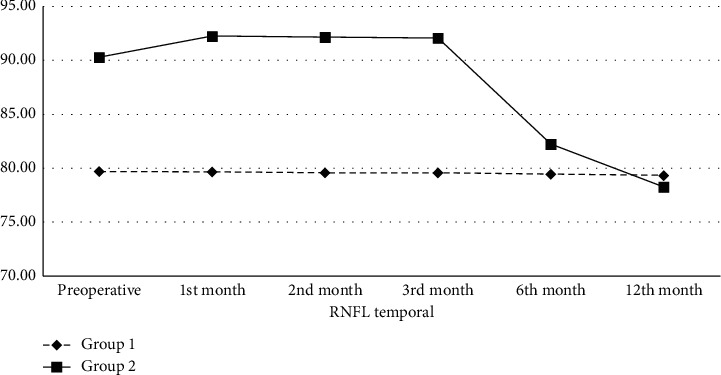
Retinal nerve fiber layer thickness (*μ*m) change in the temporal quadrant of the groups. RNFL: retinal nerve fiber layer; *μ*m: micrometer.

In the preoperative period, the mean RNFL thickness in the temporal quadrant in Group 1 and Group 2 was 79.69 ± 3.67 and 90.31 ± 3.50 *μ*m, respectively. The mean RNFL thickness in the temporal quadrant in Group 2 was statistically significantly higher than in Group 1 preoperatively (*p* < 0.01). While there was no statistically significant change in Group 1 during the follow-up period, the mean RNFL thickness in the temporal quadrant in Group 2 compared to the preoperative period was higher in the 1^st^ (92.26 ± 3.26 *μ*m, *p* < 0.01), 2^nd^ (92.18 ± 3.25 *μ*m, *p* < 0.01), and 3^rd^ (92.07 ± 3.29 *μ*m, *p* < 0.01) and lower in the 6^th^ (82.23 ± 3.57 *μ*m, *p* < 0.01) month and 12^th^ (78.25 ± 3.01 *μ*m, *p* < 0.01) month (Table 4, Figure 4).

## 4. Discussion

The diagnosis of PVD has traditionally been based on dynamic B-scan ultrasound, clinical biomicroscopy, or intraoperative evidence. Recently, OCT has been used to standardize the diagnosis of PVD [12]. High-resolution spectral-domain OCT and wide-field scanning patterns are helpful for reliably diagnosing PVD [13]. The clinical diagnosis of PVD is made by observing the Weiss ring formed by tearing the epipapillary glial tissue from the optic nerve head in a funduscopic examination [14]. In our study, we diagnosed the presence or absence of PVD with the help of biomicroscopy, B-scan ultrasonography, and OCT.

Studies investigating the effect of PPV on RNFL have also been conducted [8–11]. Gharbiya et al. investigated the effect of uncomplicated macular surgery for ERM on RNFL with OCT and reported that significant RNFL thinning in the temporal and inferior quadrants was correlated with the change in BCVA, and therefore, they hypothesized that these effects were associated with inner retinal damage [8]. Lee et al. investigated the effect of ERM on peripapillary RNFL thickness measurements using OCT and reported an increase in thickness in the temporal quadrant of the RNFL in both groups and especially in cases with ERM that were extending the peripapillary area [9]. Kim et al. conducted a similar study in patients with glaucoma and reported a statistically significant increased thickness in the RNFL, especially in the temporal quadrant [10]. Oh et al. investigated configurations of the optic nerve head (ONH) and peripapillary RNFL in eyes with ERM and reported no significant differences in ONH and RNFL parameters in eyes without retinal distortion, but increased RNFL thickness in the temporal quadrant of the eyes with retinal distortion [11]. These findings suggest that the thickening of the temporal quadrant may depend on the possible traction of the ERM. In our study, in the preoperative period, although it was more prominent in the temporal quadrant, the mean RNFL thickness in the eyes with idiopathic ERM was statistically significantly higher in the temporal, superior, and inferior quadrants than fellow eyes. There was no statistically significant difference between the groups in the nasal quadrant.

In a 12-month observational study, Lim et al. analyzed longitudinal changes in the thicknesses of the macula, ganglion cell-inner plexiform layer (GC-IPL), and peripapillary RNFL after PPV. They included 38 patients diagnosed with intraocular lens dislocation without evidence of other vitreoretinal diseases into their study and reported no significant differences in central macular thickness, increased GC-IPL thickness 1 month after surgery, and increased RNFL thickness from baseline at 1 month and 3 months after PPV. They also reported that GC-IPL returned to its preoperative thickness after 1 month and RNFL after 3 months [15]. These findings may support that not only the ERM but also PVD induced during PPV could cause transient thickness increase in the RNFL. In our study, after surgically induced PVD, the mean RNFL thickness in the nasal quadrant in the first 3 months and in the other quadrants in the first six months was larger than in the preoperative period. The increases were more noticeable in the first month. This may be a temporary result of tractional power that could develop on the RNFL during PVD induction.

In a comparative study, Mariotti et al. investigated the changes in postoperative peripapillary RNFL thickness after vitrectomy for ERM in eyes with preexisting or surgically induced PVD. They reported significant RNFL thickness decreases in temporal inferior and nasal inferior sectors in the group with surgically induced PVD, but no change except temporal sector thinning in the group with preexisting PVD [16]. Scupola et al. analyzed the relationship between swelling of the arcuate nerve fiber layer and long-term decrease of RNFL thickness after internal limiting membrane peeling for idiopathic ERM and reported decrease in the temporal sectors, and the linear extent of swelling was significantly correlated with the percentage of reduction in RNFL in the temporal and inferotemporal sectors at 12 months of follow-up [17]. Ohta et al. investigated circumpapillary RNFL thickness following macular hole (MH) surgery with ILM peeling combined with phacoemulsification and reported significant increase in the superior-temporal and superior-nasal sectors that remained until the 12^th^ month [18]. Hibi et al. analyzed the changes in the thickness of RNFL after surgery for idiopathic MH using OCT and reported significant RNFL thickness increase at 1st month postoperatively that returned to the presurgery level at 3 and 6 months. Their study indicated that the transient increase of RNFL thickness at the 1^st^ month after surgery was statistically significant in the superior, nasal, and inferior quadrants [19]. Lee et al. investigated longitudinal changes in RNFL thickness following vitrectomy for ERM. They reported that postoperative RNFL thickness tended to decrease postoperatively, and temporal quadrant RNFL thickness was statistically significantly larger in affected eyes at baseline and at 1 month after surgery and smaller after 12 months than in fellow eyes [20]. In a retrospective study on 44 patients, Kim et al. investigated RNFL change after PPV, and reported that thickness was reduced in the inferior quadrant of the vitrectomized eye for MH during the 6-month postoperative follow-up period [21]. In our study, the mean RNFL thickness at the 12^th^ month was statistically significantly lower in all quadrants except the superior compared to the preoperative period. This may be due to surgical intervention or damage to the retinal nerve fiber that may develop during the ILM peeling process.

LOH after vitrectomy may also be the cause of the changes in the RNFL. In a multicenter study on 6,048 eyes, Reibaldi et al. investigated the risk and incidence of LOH after vitrectomy. As a result of their studies, they reported that the risk of LOH was higher in vitrectomized eyes (4.9%) compared to fellow eyes (1.4%), and the main risk factors were the use of triamcinolone and postvitrectomy pseudophakic/aphakic status [22]. None of the eyes included in our study developed LOH during the one-year follow-up period after PPV. The low number of cases in our study and absence of triamcinolone use may have made it difficult for us to evaluate this important parameter.

The materials used during macular surgery that increase the visibility of the membranes may also affect RNFL thickness. In an randomized controlled trial, Arora et al. evaluated RNFL thickness after conventional BB-assisted MH surgery versus BB selective staining using autologous heparinized whole blood (WB) previously in MH surgery and reported statistically significant decrease in RNFL thickness in both groups, which was more evident in the group that received only BB [23]. Toba et al. compared the postoperative changes of RNFL thickness in patients with MH treated with PPV with indocyanine green, brilliant blue, or triamcinolone acetonide-assisted ILM peeling and reported transient increase of RNFL thickness at 1 month after surgery in all groups that returned to the baseline level in all sectors except for the nasal/inferior sector. They also reported that the differences in RNFL thickness among the groups were not significant for, at least, 12 months postoperatively [24]. Yamashita et al., in a retrospective study, analyzed RNFL thickness in eyes with or without visual field defects after indocyanine green-assisted vitrectomy for idiopathic MH using OCT and investigated the relationship between postoperative visual field defects and RNFL damage. They suggested that visual field defects may have been caused by RNFL damage relating to the use of indocyanine green [25]. Ando et al. also reported similar results in their previously published study [26]. In this study, we used BB in all cases in Group 2 for ILM peeling.

## 5. Conclusions

In conclusion, increased thickness in the RNFL may depend on the traction the ERM could form on the optic nerve. The cause of the changes in the RNFL may also be due to parameters related to vitreoretinal surgery or intraocular pressure that we did not follow in our study. Controlled, prospective, and large series studies are needed to determine the effect of these parameters on the RNFL.

## Figures and Tables

**Table 1:** Retinal nerve fiber layer thickness (*μ*m) change in the superior quadrant of the groups.

	Group 1 (fellow eyes)		Group 2 (vitrectomized eyes)		*p*
	Mean ± SD	Median	Mean ± SD	Median	
RNFL superior					
Preoperative	140.85 ± 5.86	141.05	145.89 ± 5.88	145.90	0.000^*w*^
1^st^ month	140.84 ± 5.84	141.05	150.76 ± 6.31	150.90	0.000^*w*^
Preoperative-1^st^ month change	−0.01 ± 0.13	0.00	4.87 ± 1.37	4.80	0.000^*w*^
Intergroup change *p*	0.512^*w*^		0.000^*w*^		

2^nd^ month	140.86 ± 5.88	141.00	150.88 ± 6.38	150.70	0.000^*w*^
Preoperative-2^nd^ month change	0.01 ± 0.12	0.00	4.98 ± 1.36	4.85	0.000^*w*^
Intergroup change *p*	0.417^*w*^		0.000^*w*^		

3^rd^ month	140.82 ± 5.87	141.00	150.05 ± 6.30	150.15	0.000^*w*^
Preoperative-3^rd^ month change	−0.03 ± 0.11	0.00	4.16 ± 1.69	4.10	0.000^*w*^
Intergroup change *p*	0.269^*w*^		0.000^*w*^		

6^th^ month	140.88 ± 5.86	141.10	147.20 ± 6.83	147.70	0.000^*w*^
Preoperative-6^th^ month change	0.03 ± 0.19	0.00	1.31 ± 2.18	1.20	0.003^*w*^
Intergroup change *p*	0.272^*w*^		0.000^*w*^		

12^th^ month	140.80 ± 5.84	141.05	143.35 ± 7.13	143.65	0.000^*w*^
Preoperative-12^th^ month change	−0.05 ± 0.16	0.00	−2.54 ± 2.73	−2.40	0.000^*w*^
Intergroup change *p*	0.093^*w*^		0.000^*w*^		

^*w*^Wilcoxon test, RNFL- retinal nerve fiber layer, *μ*m- micrometer, SD- standard deviation.

**Table 2:** Retinal nerve fiber layer thickness (*μ*m) change in the nasal quadrant of the groups.

	Group 1 (fellow eyes)		Group 2 (vitrectomized eyes)		*p*
	Mean ± SD	Median	Mean ± SD	Median	
RNFL nasal					
Preoperative	90.27 ± 4.69	90.45	90.22 ± 4.43	90.35	0.810^*w*^
1^st^ month	90.27 ± 4.69	90.40	93.37 ± 4.60	93.50	0.000^*w*^
Preoperative-1^st^ month change	0.00 ± 0.05	0.00	3.15 ± 1.12	3.05	0.000^*w*^
Intergroup change *p*	0.691^*w*^		0.000^*w*^		

2^nd^ month	90.26 ± 4.72	90.30	93.44 ± 4.60	93.25	0.000^*w*^
Preoperative-2^nd^ month change	−0.01 ± 0.09	0.00	3.22 ± 1.15	3.35	0.000^*w*^
Intergroup change *p*	0.332^*w*^		0.000^*w*^		

3^rd^ month	90.28 ± 4.71	90.35	92.18 ± 4.60	92.10	0.000^*w*^
Preoperative-3^rd^ month change	0.01 ± 0.10	−0.05	1.95 ± 1.29	2.00	0.000^*w*^
Intergroup change *p*	0.416^*w*^		0.000^*w*^		

6^th^ month	90.27 ± 4.72	90.25	90.30 ± 4.38	89.85	0.814^*w*^
Preoperative-6^th^ month change	0.00 ± 0.11	−0.10	0.07 ± 1.63	0.20	0.445^*w*^
Intergroup change *p*	0.512^*w*^		0.695^*w*^		

12^th^ month	90.25 ± 4.64	90.25	89.25 ± 4.38	89.20	0.067^*w*^
Preoperative-12^th^ month change	−0.02 ± 0.20	−0.20	−0.98 ± 1.87	−1.15	0.028^*w*^
Intergroup change *p*	0.264^*w*^		0.009^*w*^		

^*w*^Wilcoxon test, RNFL- retinal nerve fiber layer, *μ*m- micrometer, SD- standard deviation.

**Table 3:** Retinal nerve fiber layer thickness (*μ*m) change in the inferior quadrant of the groups.

	Group 1 (fellow eyes)		Group 2 (vitrectomized eyes)		*p*
	Mean ± SD	Median	Mean ± SD	Median	
RNFL inferior					
Preoperative	142.03 ± 6.26	141.40	145.36 ± 6.11	144.80	0.000^*w*^
1^st^ month	142.03 ± 6.31	141.50	157.15 ± 6.95	156.05	0.000^*w*^
Preoperative-1^st^ month change	0.00 ± 0.24	0.00	11.79 ± 1.83	11.85	0.000^*w*^
Intergroup change *p*	0.648^*w*^		0.000^*w*^		

2^nd^ month	142.08 ± 6.34	141.70	159.01 ± 7.13	158.00	0.000^*w*^
Preoperative-2^nd^ month change	0.05 ± 0.58	0.00	13.65 ± 2.17	13.55	0.000^*w*^
Intergroup change *p*	0.128^*w*^		0.000^*w*^		

3^rd^ month	142.03 ± 6.33	141.40	159.41 ± 7.10	159.10	0.000^*w*^
Preoperative-3^rd^ month change	0.00 ± 0.30	0.00	14.05 ± 2.35	13.65	0.000^*w*^
Intergroup change *p*	0.517^*w*^		0.000^*w*^		

6^th^ month	142.04 ± 6.27	141.40	150.10 ± 8.48	150.20	0.000^*w*^
Preoperative-6^th^ month change	0.01 ± 0.36	−0.05	4.73 ± 4.17	5.10	0.000^*w*^
Intergroup change *p*	0.412^*w*^		0.000^*w*^		

12^th^ month	142.06 ± 6.31	141.20	139.67 ± 6.96	138.95	0.020^*w*^
Preoperative-12^th^ month change	0.03 ± 0.40	−0.10	−5.69 ± 3.58	−5.35	0.000^*w*^
Intergroup change *p*	0.263^*w*^		0.000^*w*^		

^*w*^Wilcoxon test, RNFL- retinal nerve fiber layer, *μ*m- micrometer, SD- standard deviation.

**Table 4:** Retinal nerve fiber layer thickness (*μ*m) change in the temporal quadrant of the groups.

	Group 1 (fellow eyes)		Group 2 (vitrectomized eyes)		*p*
	Mean ± SD	Median	Mean ± SD	Median	
RNFL temporal					
Preoperative	79.69 ± 3.67	79.60	90.31 ± 3.50	90.05	0.000^*w*^
1^st^ month	79.69 ± 3.66	79.55	92.26 ± 3.26	91.80	0.000^*w*^
Preoperative-1^st^ month change	0.00 ± 0.07	0.00	1.95 ± 1.06	2.05	0.000^*w*^
Intergroup change *p*	0.315^*w*^		0.000^*w*^		

2^nd^ month	79.68 ± 3.71	79.40	92.18 ± 3.25	91.65	0.000^*w*^
Preoperative-2^nd^ month change	−0.01 ± 0.21	−0.10	1.86 ± 1.05	2.00	0.000^*w*^
Intergroup change *p*	0.198^*w*^		0.000^*w*^		

3^rd^ month	79.67 ± 3.70	79.40	92.07 ± 3.29	91.50	0.000^*w*^
Preoperative-3^rd^ month change	−0.02 ± 0.27	−0.10	1.76 ± 1.03	1.90	0.000^*w*^
Intergroup change *p*	0.098^*w*^		0.000^*w*^		

6^th^ month	79.71 ± 3.66	79.40	82.23 ± 3.57	81.55	0.000^*w*^
Preoperative-6^th^ month change	0.02 ± 0.32	−0.20	−8.09 ± 2.59	−8.25	0.000^*w*^
Intergroup change *p*	0.075^*w*^		0.000^*w*^		

12^th^ month	79.70 ± 3.65	79.35	78.25 ± 3.01	78.50	0.032^*w*^
Preoperative-12^th^ month change	0.01 ± 0.39	−0.30	−12.06 ± 2.99	−12.30	0.000^*w*^
Intergroup change *p*	0.216^*w*^		0.000^*w*^		

^*w*^Wilcoxon test, RNFL- retinal nerve fiber layer, *μ*m- micrometer, SD- standard deviation.

## Data Availability

The data produced and analyzed during the current study are not publicly available due to the prohibition of the hospital's archive system, but could be obtained from the corresponding author upon plausible and acceptable request.

## Additional Points

Our study has some limitations. Firstly, the study was conducted on a small number of cases. Secondly, the severity of the ERM was not classified preoperatively. In addition, it may not be possible to determine whether PVD induction caused such changes in the RNLF because other surgical and clinical variables may have determined it.
